# Preliminary Immunogenicity Evaluation of an Immunoinformatics-Guided Multi-Epitope mRNA Vaccine Against Porcine Epidemic Diarrhea Virus

**DOI:** 10.3390/vaccines14050388

**Published:** 2026-04-27

**Authors:** Yiqing Liu, Huanhui Huang, Ya Chen, Jianhong Shu, Fangli Wu

**Affiliations:** 1 Key Laboratory of Plant Secondary Metabolism and Regulation of Zhejiang Province, College of Life Sciences and Medicine, Zhejiang Sci-Tech University, Hangzhou 310018, China; 2023210901042@mails.zstu.edu.cn (Y.L.); hhh00081919@163.com (H.H.); 2023210901009@mails.zstu.edu.cn (Y.C.); shujianhong@zstu.edu.cn (J.S.); 2 Zhejiang Sci-Tech University Shaoxing Academy of Biomedicine, Shaoxing 312366, China

**Keywords:** porcine epidemic diarrhea virus (PEDV), mRNA vaccine, multi-epitope design, immunoinformatics, lipid nanoparticles, immunogenicity

## Abstract

**Background:** Porcine epidemic diarrhea virus (PEDV) remains a major threat to the global swine industry, highlighting the urgent need for safe and effective next-generation vaccines. mRNA vaccines have emerged as a promising platform due to their rapid development and favorable safety profile. **Objectives:** This study aimed to design and perform the preliminary evaluation of a PEDV multi-epitope mRNA vaccine using an immunoinformatics-guided strategy combined with experimental validation. **Methods:** Immunoinformatics tools were used to identify B-cell and cytotoxic T lymphocyte (CTL) epitopes from the PEDV spike (S), membrane (M), and nucleocapsid (N) proteins. Selected epitopes were assembled into a multi-epitope antigen (E). mRNA constructs encoding S1, S2, and antigen E were synthesized via in vitro transcription and encapsulated into lipid nanoparticles (LNPs). Expression was evaluated in HEK293T cells, and immunogenicity was assessed in mice measuring antigen-specific antibody responses and cytokine levels following immunization. **Results:** The mRNA constructs exhibited high structural integrity and efficient intracellular translation. The LNP formulations showed good physicochemical stability and delivery efficiency. Immunization with the antigen E mRNA-LNP formulation induced significantly higher PEDV-specific IgG levels compared with control groups. Elevated cytokine levels further indicated activation of both humoral and cellular immune responses. **Conclusions:** This study presents a feasible workflow for the development of a PEDV multi-epitope mRNA vaccine. The antigen E construct demonstrated favorable immunogenicity in a mouse model, supporting its potential as a promising construct for further investigation and optimization. Although further studies are required to validate antigen expression at the protein level and to further characterize immune mechanisms, these findings provide preliminary evidence supporting the feasibility of multi-epitope mRNA vaccines for PEDV prevention.

## 1. Introduction

Porcine epidemic diarrhea virus (PEDV) is a highly contagious coronavirus that causes severe gastrointestinal disease in pigs, particularly in neonatal piglets, leading to substantial economic losses worldwide [1,2,3,4,5,6]. Clinical symptoms include acute watery diarrhea, vomiting, and dehydration [3]. Although conventional vaccines, such as inactivated and attenuated vaccines, have been widely used, their protective efficacy against emerging PEDV variants remains limited [7,8]. Therefore, the development of more effective vaccine strategies is urgently needed [9,10].

One major challenge is the extensive genetic diversity of PEDV, particularly within the spike (S) gene, which plays a key role in viral entry and immune recognition. Antigenic variation in critical neutralizing epitopes contributes to immune escape and reduced cross-protection, thereby compromising vaccine efficacy [11,12]. These findings highlight the need for next-generation vaccine strategies capable of inducing broader and more durable immune responses.

mRNA vaccine technology has demonstrated great promise due to its rapid design [13], flexible production [14], and strong immunogenicity [15,16], as exemplified by its successful application during the COVID-19 pandemic [17,18]. Delivered via lipid nanoparticles (LNPs), mRNA vaccines efficiently express target antigens and induce both humoral and cellular immune responses [19,20]. Importantly, mRNA technology allows precise antigen design, including multi-epitope chimeric constructs that broaden and prolong immune protection [21,22]. These advantages make mRNA vaccines particularly suitable for combating highly variable viruses such as PEDV, where antigenic diversity poses a major challenge for conventional vaccine strategies. Recent advances have further emphasized the value of immunoinformatics-guided multi-epitope design as an effective strategy to overcome antigenic variability by integrating multiple conserved B- and T-cell epitopes [23], particularly for rapidly evolving viruses such as coronaviruses [24,25].

The PEDV spike (S) protein is a key structural glycoprotein responsible for viral entry into host cells and serves as a primary target for vaccine development. It mediates viral attachment to host cell receptors and subsequent membrane fusion, making it a critical determinant of viral infectivity and host immune recognition [26]. Due to its large size and complex structure, the full-length S protein is technically challenging to express. During viral entry, the S protein is cleaved by host proteases into two functional subunits, S1 and S2, and it is therefore commonly divided into these subunits for antigen design. The S1 subunit is primarily responsible for receptor binding, whereas the S2 subunit mediates membrane fusion. Structural studies have further revealed that conformational changes in the S protein are essential for viral entry [26]. The S1 subunit contains several well-characterized neutralizing epitopes, including the N-terminal domain (NTD), COE domain, and linear neutralizing regions, making it an attractive vaccine target [27]. Antibodies targeting specific regions of the S protein, such as the COE domain, have been shown to effectively neutralize PEDV and prevent infection [28]. In addition, mutations within the S gene, particularly in receptor-binding regions, have been associated with antigenic variation and differences in virulence among PEDV strains, further complicating vaccine design [29]. However, vaccines based on a single epitope may be limited by epitope masking or immune escape [30,31]. In contrast, multi-epitope vaccine designs that combine multiple conserved neutralizing regions may enhance cross-protective immunity against diverse PEDV strains [25,32].

Recent studies have confirmed the feasibility of PEDV mRNA vaccines [33]. For example, Zhao et al. developed an mRNA-LNP vaccine encoding the full-length S protein and a multi-epitope chimeric Sm protein, which induced strong neutralizing antibody responses and PEDV-specific T-cell immunity in piglets, leading to reduced viral loads [19].

Despite these advances, current PEDV mRNA vaccine strategies remain limited by suboptimal antigen selection and insufficient breadth of immune protection against diverse viral variants. In particular, most existing studies have primarily focused on the spike (S) protein, which, although capable of inducing neutralizing antibodies, has shown limited cross-protective efficacy against emerging PEDV variants [34]. In addition, certain regions within the S protein have been reported to contain enhancing epitopes that may induce non-neutralizing antibodies, potentially affecting immune effectiveness [35].

Therefore, in this study, we designed and preliminarily evaluated a multi-epitope mRNA vaccine candidate integrating epitopes derived from multiple structural proteins (S, M, and N) based on immunoinformatics-guided antigen selection, with the aim of enhancing cross-protective immunity and improving antigen coverage. Compared with previously reported PEDV vaccine strategies, this approach provides a proof-of-concept framework for multi-antigen and multi-epitope vaccine design, which may contribute to improving immune breadth and addressing viral diversity in future studies.

## 2. Materials and Methods

### 2.1. Immunoinformatic Design of the Multi-Epitope Antigen

The complete proteome sequence of the Porcine Epidemic Diarrhea Virus (PEDV, CV777 strain) was retrieved from the ViralZone Expasy database and used for multi-epitope antigen design through an immunoinformatic approach [36].

Antigenicity analysis of the PEDV proteome was performed using VaxiJen 2.0 and ANTIGENpro 1.0, with a cutoff value of 0.4. VaxiJen was selected as an alignment-independent prediction tool based on physicochemical properties, while ANTIGENpro employs a sequence-based machine learning approach, providing complementary evaluation of antigenicity. These complementary approaches were combined to improve prediction robustness and reduce method-specific bias. The cutoff value of 0.4 was selected based on commonly adopted thresholds for viral antigen prediction reported in previous studies [37,38]. Prediction of linear B-cell epitopes was performed using both ABCpred 1.0 (threshold > 0.8) and BepiPred 2.0 available from the Immune Epitope Database (IEDB) [39]. ABCpred is based on artificial neural networks, whereas BepiPred 2.0 integrates a random forest algorithm trained on epitope datasets, allowing complementary prediction performance. The threshold of 0.8 for ABCpred was selected to improve specificity, as higher thresholds reduce false-positive predictions. A stringent threshold of 0.8 was applied to ABCpred to increase prediction specificity and minimize false-positive results, in accordance with previous epitope prediction studies. Only the overlapping regions identified by both algorithms were retained as potential B-cell epitopes. To further assess the spatial accessibility of candidate antigens, three-dimensional structural models were generated using the SWISS-MODEL server based on the corresponding amino acid sequences [40], enabling evaluation of surface accessibility, which is essential for antibody recognition. CTL epitopes were predicted using NetMHCpan 4.1 based on porcine SLA-1 alleles (SLA-1:0101, SLA-1:0401, SLA-1:0801) with a binding affinity cutoff of 0.5 [41,42]. NetMHCpan 4.1 is a widely validated tool for predicting peptide–MHC binding prediction. The selected SLA alleles represent commonly distributed porcine MHC class I molecules, improving biological relevance. A strict affinity threshold was applied to retain peptides with strong binding potential, following established immunoinformatics practices for CTL epitope screening.

Selected epitopes were concatenated using flexible linkers to construct the final multi-epitope antigen (designated as E). B-cell epitopes were joined by KK linkers [42], while T-cell epitopes were connected by AAY linkers [43]. A tPA signal peptide was incorporated at the N-terminus to promote protein secretion, and a T4 phage Foldon trimerization domain was fused to the C-terminus to enhance structural stability and immunogenicity. Two (GGGGS) linkers were inserted between adjacent antigenic components to maintain conformational flexibility [44].

**Figure 1:**
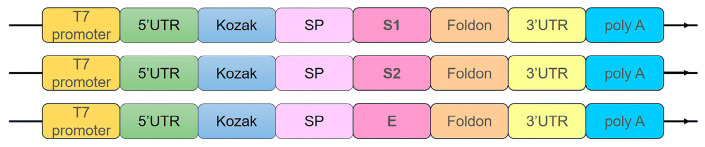
Schematic representation of the PEDV mRNA vaccine expression cassette. Three mRNA constructs share an identical backbone comprising a T7 promoter, 5′ and 3′ untranslated regions (UTRs), a Kozak sequence, a signal peptide (SP), a foldon domain, and a poly(A) tail. The constructs differ only in the antigen-coding region, which encodes the PEDV S1, S2, or a rationally designed E protein, respectively.

Codon optimization was subsequently carried out for both murine and porcine expression systems, followed by commercial gene synthesis. The optimized E sequence, along with the S1 and S2 antigen sequences, was cloned into a pUC-19 vector containing a T7 promoter, 5′/3′ UTRs, and a poly(A) tail to establish the complete mRNA vaccine expression cassettes [45]. These constructs served as templates for subsequent in vitro transcription (IVT) reactions (Figure 1).

### 2.2. mRNA Vaccine Construct Preparation

Recombinant plasmids were generated by homologous recombination. Positive clones were identified by antibiotic selection and molecular verification, and endotoxin-free plasmid DNA was purified for downstream applications. Capped mRNA transcripts were synthesized using an EasyCap T7 (Vazyme, Nanjing, China) co-transcription kit with linearized plasmids as templates, followed by purification and quantification. Purified mRNA was subsequently encapsulated into lipid nanoparticles (LNPs) using a commercial transfection kit (HuiXin Bio, Hangzhou, China) and used directly for in vitro or in vivo applications.

### 2.3. Particle Size and Polydispersity Index (PDI) Measurement

The hydrodynamic diameter and PDI of the mRNA–LNP formulations were determined by dynamic light scattering (DLS). Samples were diluted with RNase-free water to 1 mL, gently mixed, and measured using a nano-particle size analyzer (Malvern, UK) at room temperature.

### 2.4. Encapsulation Efficiency Assay

mRNA encapsulation efficiency was determined using the Quant-iT™ RiboGreen™ RNA assay kit (Thermo Fisher Scientific, Waltham, MA, USA) following the manufacturer’s instructions. Total RNA was measured after LNP lysis, and free RNA was measured in intact LNPs. Encapsulation efficiency was calculated as the percentage of RNA protected within LNPs using the formula: Encapsulation (%) = [1 − (free RNA/total RNA)] × 100.

### 2.5. In Vitro Expression of mRNA

HEK293T cells were cultured in high-glucose DMEM supplemented with 10% fetal bovine serum and 1% penicillin–streptomycin at 37 °C with 5% CO_2_. Cells were seeded in six-well plates and used at ~80% confluency. To assess mRNA–LNP delivery efficiency, eGFP–mRNA–LNPs were employed as reporter formulations. Cells were transfected with naked eGFP–mRNA or eGFP–mRNA–LNPs, and eGFP expression was evaluated by fluorescence microscopy 24 h post-transfection.

### 2.6. Animals and Experimental Design

**Figure 2:**
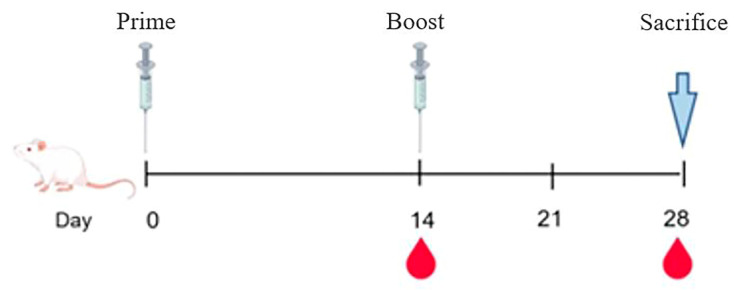
Schematic diagram of the mouse immunization and safety evaluation process. Mice were primed on day 0. On day 14, submandibular blood was collected prior to booster immunization. A second blood sample was collected on day 28, after which the mice were euthanized for downstream analyses.

SPF BALB/c mice (6–8 weeks old; *n* = 20, Hangzhou Hans Biotechnology Co., Ltd. Hangzhou, China) were randomly assigned to control or vaccine groups. Mice were immunized intramuscularly with mRNA-LNP vaccines or controls, boosted after two weeks, and monitored for health. Blood samples were collected at defined time points, and mice were euthanized following the final collection (Figure 2). All procedures were approved by the institutional animal ethics committee.

### 2.7. Animal Ethics Statement

All animal experiments were conducted in strict accordance with the Guidelines for the Care and Use of Laboratory Animals. The experimental protocols were reviewed and approved by the Institutional Animal Care and Use Committee (IACUC) of Zhejiang Sci-Tech University (Approval No. 20231024-12). All efforts were made to minimize animal suffering and to reduce the number of animals used.

### 2.8. PEDV-Specific IgG ELISA

Blood samples were collected, allowed to clot, and centrifuged to obtain serum, which was stored at −20 °C until analysis. PEDV-specific IgG levels were quantified using a commercial mouse anti-PEDV IgG ELISA kit (Guyan, Shanghai, China) according to the manufacturer’s instructions. Optical density was measured at 450 nm using a microplate reader (BMG Labtech, Ortenberg, Germany).

### 2.9. Virus Titration and Neutralization Assay

The infectious titer of the PEDV stock was determined using the Reed–Muench method. Serial tenfold dilutions of the virus were added to Vero cells, and cytopathic effects (CPEs) were monitored to calculate TCID_50_. For the serum neutralization assay, heat-inactivated serum samples were serially diluted and incubated with PEDV before being added to Vero cells. Neutralizing titers (NT_50_) were defined as the highest serum dilution that inhibited CPE in 50% of wells, calculated using the Reed–Muench method.

### 2.10. Cytokine Quantification

Levels of interleukin-4 (IL-4) and interferon-gamma (IFN-γ) in mouse sera were quantified using specific ELISA kits (Ruixin Biotech, Quanzhou, China) according to the manufacturer’s instructions. Briefly, serum samples were added to pre-coated 96-well plates, incubated with biotinylated detection antibodies, and detected by HRP-conjugated streptavidin. After color development and termination, OD values were measured at 450 nm, and cytokine concentrations were calculated from standard curves.

### 2.11. Safety Evaluation

In vivo safety of the PEDV mRNA–LNP vaccines was evaluated in immunized mice by monitoring body weight and histopathological analysis. After the immunization schedule, major organs (heart, liver, and kidneys) were collected and fixed in 4% paraformaldehyde. Tissues were paraffin-embedded, sectioned, and stained with hematoxylin and eosin (H&E). Histological analysis was performed under blinded conditions by Hangzhou Hulk Biotechnology Co., Ltd. (Hangzhou, China).

### 2.12. Statistical Analysis

All statistical analyses and graphical visualizations were performed using GraphPad Prism version 10.1.2 (GraphPad Software, San Diego, CA, USA). Data are presented as mean ± standard deviation (SD) unless otherwise specified.

Comparisons among multiple groups were conducted using one-way analysis of variance (ANOVA) followed by Tukey’s multiple comparison test to determine statistical significance. The level of significance was set as follows: *** *p* < 0.001, ** *p* < 0.01, * *p* < 0.05, while ns denotes no statistically significant difference.

Statistical differences between treatment groups were indicated by asterisks or distinct lettering in figures, as specified in figure legends.

## 3. Results

### 3.1. Design and In Silico Validation of the Novel PEDV Multi-Epitope Antigen

To screen and prioritize potential candidate antigens, antigenicity prediction was performed using both VaxiJen and ANTIGENpro. VaxiJen analysis indicated that all evaluated PEDV structural proteins exceeded the threshold score of 0.4, suggesting overall antigenic potential, whereas ANTIGENpro analysis revealed that only the spike (S) and nucleocapsid (N) proteins achieved antigenicity scores above the same cutoff (Table 1). Based on these combined results, the spike (S) and nucleocapsid (N) proteins were prioritized and selected as the primary candidate antigens.

The membrane (M) protein did not rank among the top candidates according to antigenicity scores, published evidence suggests that it harbors several immunodominant B-cell epitopes [46]. Accordingly, the M protein was also selected as a candidate antigen for further evaluation.

### 3.2. B-Cell Epitope Prediction

To identify high-confidence linear B-cell epitopes suitable for vaccine design, candidate epitopes were screened based on sequence features; however, no linear B-cell epitopes meeting the predefined criteria were detected within the nucleocapsid (N) protein sequence. Therefore, the N protein was excluded from subsequent B-cell epitope analysis. Ultimately, seven high-confidence linear B-cell epitopes were selected, including five on the spike (S) protein and two on the membrane (M) protein. Antigenicity evaluation using VaxiJen (threshold: 0.4) confirmed that all selected epitopes exhibited acceptable antigenic potential (Table 2).

**Figure 3:**
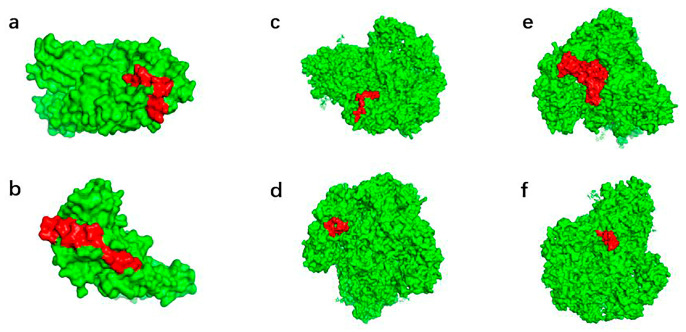
Structural mapping of predicted linear B-cell epitopes on viral proteins. Epitope regions on the target protein are highlighted in red. (**a**–**d**) Structural positions of the selected linear B-cell epitopes on the spike (S) protein. (**e**,**f**) Structural positions of the selected linear B-cell epitopes on the membrane (M) protein.

To further assess their spatial accessibility, three-dimensional structural models of the spike (S) protein and membrane (M) protein were generated using the SWISS-MODEL server based on their corresponding amino acid sequences. The selected linear B-cell epitopes were subsequently mapped onto the predicted protein structures, with epitope regions highlighted for visualization. Structural analysis indicated that the retained epitopes were predominantly distributed on surface-exposed regions of the modeled proteins, supporting their potential accessibility to B-cell receptors (Figure 3). Notably, the C-terminal region of the spike protein beyond residue 1254 could not be reliably modeled due to the lack of suitable structural templates and was therefore excluded from the epitope mapping analysis.

### 3.3. T-Cell Epitope Prediction

To identify potential cytotoxic T lymphocyte (CTL) epitopes with strong binding affinity, CTL epitope prediction and binding affinity analysis were performed, resulting in the identification of thirteen T-cell epitopes, including seven derived from Q91AV1, three from P59771, and three from Q07499 (Table 3). All selected epitopes exhibited antigenicity scores above 0.4.

All seven epitopes derived from Q91AV1 showed strong binding affinity to the three evaluated SLA-1 alleles (SLA-1:0101, SLA-1:0401, and SLA-1:0801), suggesting a relatively broad MHC class I binding profile. In contrast, epitopes from P59771 and Q07499 displayed allele-dependent binding patterns. For P59771, the first two epitopes exhibited weak binding to SLA-1:0101 but strong binding to SLA-1:0401 and SLA-1:0801, whereas the third epitope showed the opposite trend. A similar binding preference was observed for the three epitopes derived from Q07499.

Overall, epitopes derived from Q91AV1 exhibited broader binding affinity across the evaluated SLA alleles, whereas epitopes from P59771 and Q07499 showed more allele-specific binding patterns.

### 3.4. Construction and Structural Modeling of the Multi-Epitope Antigen

**Figure 4:**
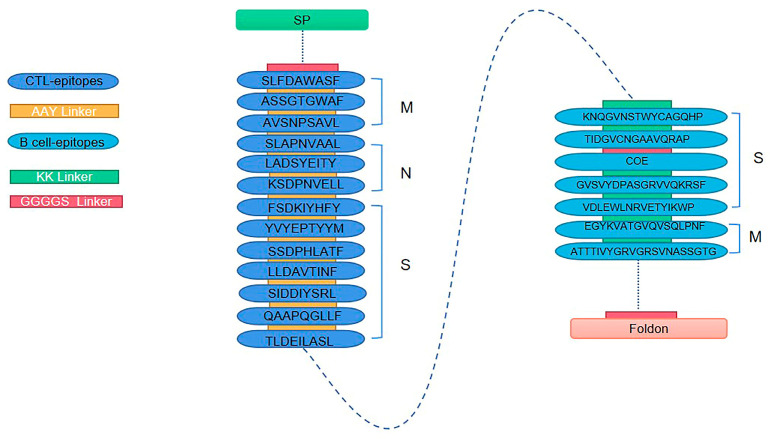
Schematic representation of the novel multi-epitope protein E, constructed by tandemly linking predicted B-cell and CTL epitopes. The construct was assembled by tandemly linking seven predicted B-cell epitopes (five from the S protein and two from the M protein) and thirteen CTL epitopes (seven from S, three from M, and three from the N protein) as described in the Methods. In the diagram, S, M, and N indicate the selected spike, membrane, and nucleocapsid protein-derived regions, respectively; SP denotes the signal peptide, and foldon represents the T4 foldon trimerization domain. Dashed lines indicate connections between protein regions. This schematic illustrates the overall arrangement of the epitopes in the designed antigen.

The predicted B-cell and CTL epitopes were assembled into a novel multi-epitope antigen, designated E, by connecting the epitopes as described in the Methods. The resulting construct is illustrated in Figure 4. This design aimed to improve the structural stability and immunogenicity of the final antigen construct.

### 3.5. Quality Evaluation of Transcribed mRNA and mRNA–LNPs

**Figure 5:**
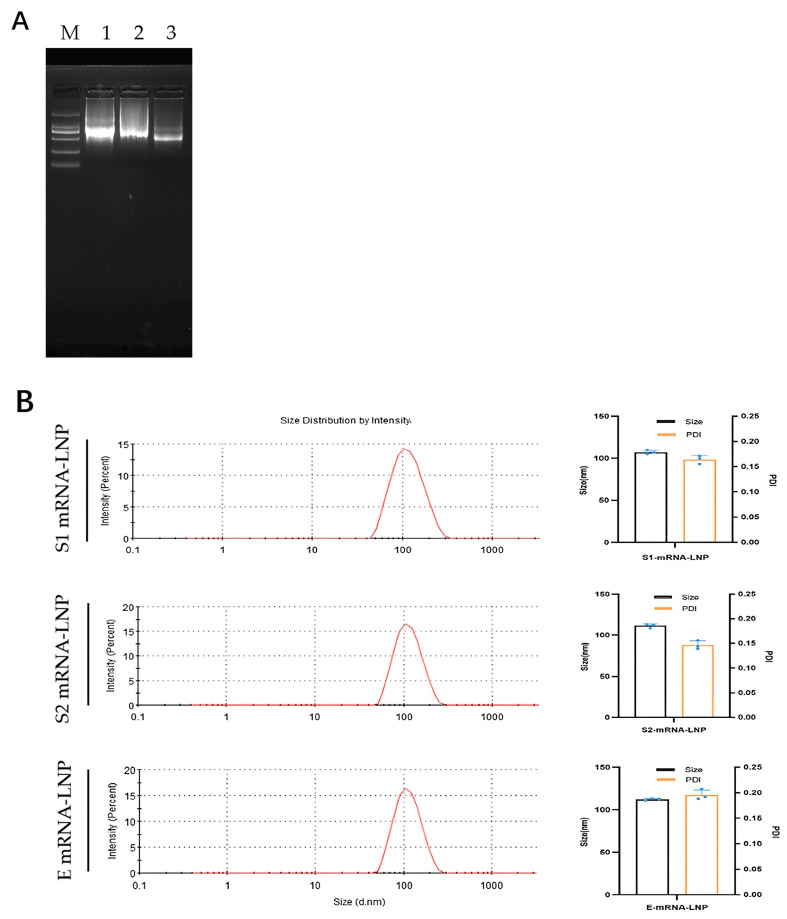
Preparation and physicochemical characterization of mRNA and mRNA–LNP formulations. (**A**) Agarose gel electrophoresis analysis of in vitro-transcribed mRNA encoding S1, S2, and E antigens. Lane M, RNA marker; lanes 1–3, S1 mRNA, S2 mRNA, and E mRNA, respectively, showing single bands of expected sizes. (**B**) Particle size distribution and polydispersity index (PDI) of mRNA–LNP formulations encapsulating S1, S2, and E mRNA, as determined by dynamic light scattering. The uncropped blots and molecular weight markers are shown in Supplementary Materials.

To evaluate the integrity and encapsulation efficiency of the synthesized mRNAs, all in vitro-transcribed mRNA samples were first analyzed, showing single, sharp bands at the expected sizes with no visible degradation, indicating high mRNA integrity (Figure 5A). These mRNAs were then encapsulated into lipid nanoparticles, and the physicochemical properties of the resulting formulations were characterized (Figure 5B). All mRNA–LNP formulations exhibited high encapsulation efficiencies (>90%), uniform particle size distributions, and low polydispersity indices (PDI < 0.2), with mean hydrodynamic diameters of 107.2 nm for mRNA–S1–LNP, 111.3 nm for mRNA–S2–LNP, and 112.2 nm for mRNA–E–LNP.

**Figure 6:**
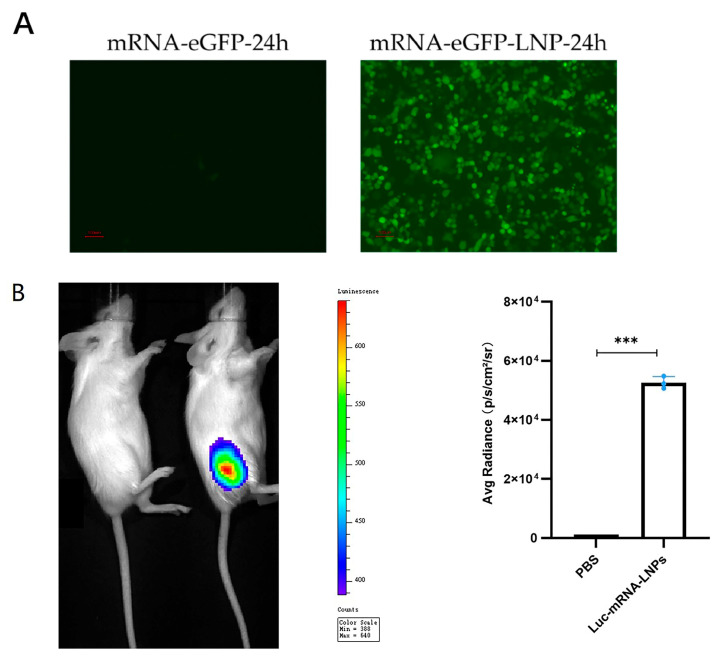
In vitro and in vivo expression validation of mRNA–LNP formulations. (**A**) In vitro expression of eGFP at 24 h post-transfection in HEK293T cells treated with naked eGFP–mRNA (control) or eGFP–mRNA–LNPs, observed by fluorescence microscopy. Strong green fluorescence signals were detected in the mRNA–LNP group, while minimal fluorescence was observed in the naked mRNA control group. (**B**) In vivo bioluminescence imaging of mice at 12 h after intramuscular injection of luciferase–mRNA–LNPs (10 μg), showing robust reporter gene expression localized at the injection site in the thigh muscle, whereas no detectable signal was observed in the PBS control group. Data are presented as mean ± SD. *** *p* < 0.001.

To assess the transfection efficiency of the LNP system in vitro, cells were transfected with eGFP–mRNA–LNPs, which exhibited strong and sustained fluorescence, whereas negligible signals were observed in the naked mRNA control (Figure 6A).

To assess in vivo delivery, Luciferase–mRNA–LNP complexes (10 μg) were administered via intramuscular injection into the right hind limb of mice, with PBS serving as the control. Bioluminescence imaging performed 12 h post-injection revealed strong luminescent signals at the injection sites of vaccinated mice, while no signal was detected in controls (Figure 6B).

### 3.6. PEDV-Specific IgG Antibody Response

**Figure 7:**
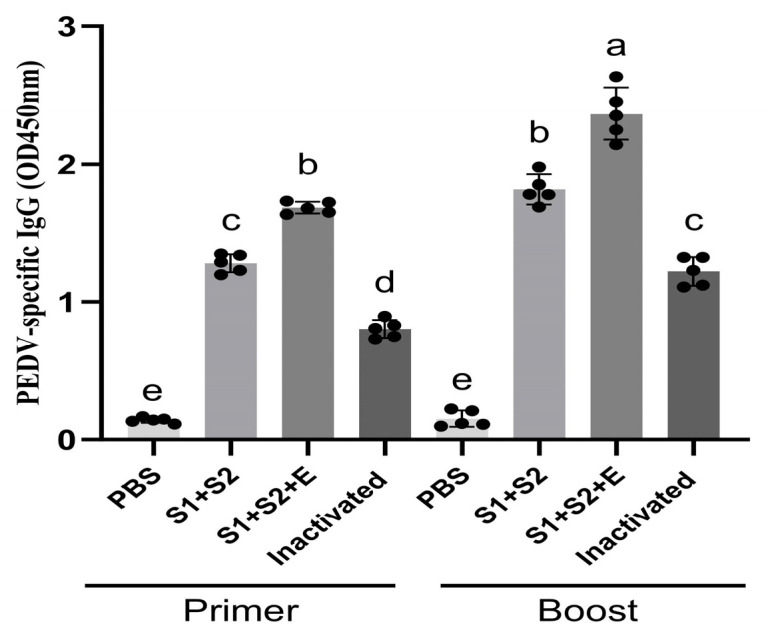
PEDV-specific IgG titers in mouse serum. Data represent mean ± SEM (n = 5). One-way ANOVA followed by Tukey’s post hoc test was used for statistical comparison. Different letters indicate statistically significant differences (*p* < 0.05), while identical letters indicate no significant difference.

To evaluate the humoral immune response induced by the PEDV mRNA vaccines, serum samples were collected on day 14 after the primary immunization and again on day 14 following the booster dose. PEDV-specific IgG levels were determined by ELISA. As shown in Figure 7, all vaccinated groups—including the inactivated vaccine, mRNA-S1+S2-LNP, and mRNA-S1+S2+E-LNP—exhibited significantly higher IgG levels compared with the PBS control group (*p* < 0.001).

After the primary immunization, the antibody level of the mRNA-S1+S2-LNP group was approximately half that of the inactivated vaccine, while the mRNA-S1+S2+E-LNP group exhibited a slightly higher level than the inactivated vaccine. Following booster immunization, both mRNA vaccine groups showed further increases, with IgG titers in the mRNA-S1+S2-LNP and mRNA-S1+S2+E-LNP groups reaching approximately 0.6-fold and 1.2-fold higher than the inactivated vaccine, respectively. In addition, all vaccinated groups were significantly higher than the PBS control group, consistent with the statistical analysis shown in Figure 7.

Comparison of antibody titers before and after booster immunization revealed that IgG levels increased by 1.39-fold in the mRNA-S1+S2-LNP group, 1.43-fold in the mRNA-S1+S2+E-LNP group, and only 0.6-fold in the inactivated vaccine group. These comparisons were based on statistically significant differences among groups as indicated in Figure 7, and no selective exclusion of comparison groups was applied. These results indicate that both mRNA vaccines effectively elicited robust humoral immune responses and that inclusion of the E epitope further enhanced antibody production and immune potency.

### 3.7. Serum Virus Neutralization Assay

**Figure 8:**
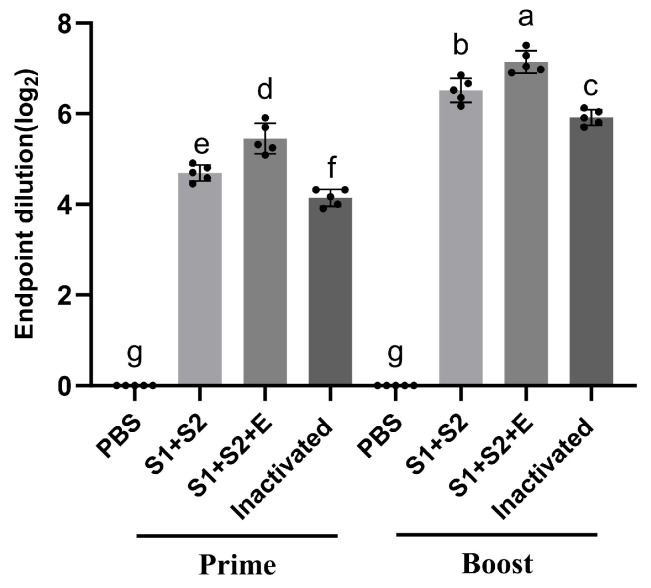
Serum neutralizing antibody titers in immunized mice. Data represent mean ± SEM (n = 5). One-way ANOVA with Tukey’s multiple comparison test was used, different letters denote significant differences (*p* < 0.05).

To evaluate the neutralizing antibody responses elicited by the vaccines, titers were determined using a live-virus neutralization assay. As illustrated in Figure 8, all vaccine groups induced potent neutralizing antibody responses (*p* < 0.01). All comparisons among groups were statistically significant as indicated by different letters in Figure 8, representing distinct significance groups determined by post hoc analysis.

After the first immunization, the neutralization titers (NT_50_) of the inactivated vaccine, mRNA-S1+S2-LNP, and mRNA-S1+S2+E-LNP groups were 1:18, 1:26, and 1:46, respectively, with the latter showing an approximately 0.77-fold increase relative to mRNA-S1+S2-LNP. These groups corresponded to distinct statistical categories as indicated by different letters, confirming significant differences among all vaccine formulations at the prime stage. Following booster immunization, neutralization titers increased substantially to 1:62, 1:94, and 1:142, respectively. The boosted groups also showed statistically significant differences among each other (c, b, a), as indicated in Figure 8. The inclusion of the E epitope resulted in an additional 0.5-fold enhancement in neutralizing activity compared with mRNA-S1+S2-LNP.

Relative to primary immunization, booster vaccination increased NT_50_ values by 2.62-fold, 2.09-fold, and 2.44-fold in the inactivated, mRNA-S1+S2-LNP, and mRNA-S1+S2+E-LNP groups, respectively. PBS control groups remained at baseline levels and were grouped separately (g) across both prime and booster stages. Collectively, these results confirm that both mRNA vaccines successfully induced high levels of neutralizing antibodies, with mRNA-S1+S2+E-LNP demonstrating superior immunogenicity and enhanced viral neutralization.

### 3.8. Cytokine Secretion Profile

**Figure 9:**
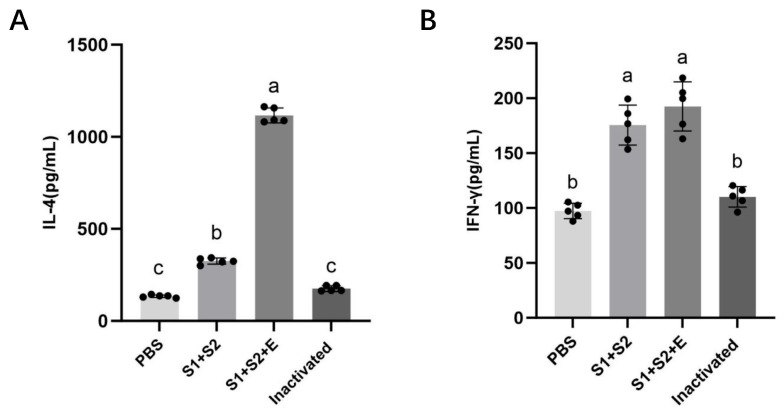
Cytokine levels in mouse sera. (**A**) IL-4; (**B**) IFN-γ. Data represent mean ± SEM (n = 5). One-way ANOVA with Tukey’s post hoc test; different letters denote significant differences (*p* < 0.05).

To further characterize the type of immune response induced, serum cytokine levels of interleukin-4 (IL-4) and interferon-gamma (IFN-γ) were quantified by ELISA (Figure 9). All vaccinated groups showed significantly higher cytokine levels compared with both the PBS and inactivated vaccine groups (*p* < 0.001), as indicated in Figure 9. Both mRNA vaccine groups exhibited significantly higher cytokine production than the PBS and inactivated vaccine groups (*p* < 0.001).

In particular, the mRNA-S1+S2+E-LNP vaccine induced an approximately 3.4-fold increase in IL-4 secretion compared with mRNA-S1+S2-LNP, suggesting a tendency toward enhanced Th2-associated responses that may support humoral immunity. This difference was statistically supported by significant separation between the two mRNA vaccine groups in IL-4 levels (Figure 9A). In contrast, IFN-γ levels did not differ significantly between the two mRNA vaccine groups (*p* > 0.05), while both groups still showed significantly higher IFN-γ levels compared with PBS and inactivated vaccine controls (Figure 9B). However, both mRNA vaccine groups still showed significantly higher IFN-γ levels compared with PBS and inactivated vaccine controls (Figure 9B).

Together, these results suggest that the inclusion of the E epitope enhances Th2-associated cytokine production, promoting stronger antibody-mediated protection. Importantly, this interpretation is based on the observed increase in IL-4 rather than a reduction in IFN-γ, indicating a shift toward a more humoral-favored immune profile rather than a polarized Th1/Th2 imbalance.

### 3.9. Flow Cytometric Analysis of Splenic T-Cell Subsets

**Figure 10:**
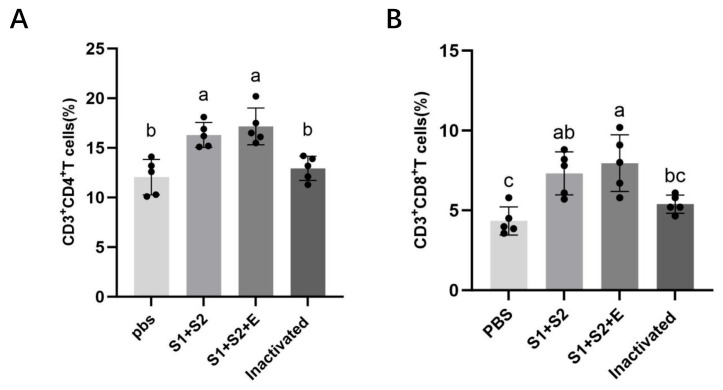
Flow cytometric analysis of splenic T-cell subsets. (**A**) Percentage of CD3^+^CD4^+^ cells; (**B**) Percentage of CD3^+^CD8^+^ cells. Data represent mean ± SEM (n = 5). One-way ANOVA with Tukey’s post hoc test; different letters denote significant differences (*p* < 0.05).

To evaluate the vaccine-induced cellular immune responses, splenocytes were isolated from immunized mice, and the proportions of CD3^+^CD4^+^ and CD3^+^CD8^+^ T cells were quantified by flow cytometry (Figure 10). Both mRNA vaccine groups showed significantly higher CD4^+^ and CD8^+^ T-cell proportions compared with both the PBS and inactivated vaccine groups (*p* < 0.05), as indicated in Figure 10.

Compared with the PBS control, both mRNA vaccine groups exhibited significant increases in CD4^+^ and CD8^+^ T-cell populations (*p* < 0.05), whereas the inactivated vaccine induced minimal changes. The CD3^+^CD4^+^ T-cell proportion increased from 12.06% (PBS) and 12.94% (inactivated) to 16.30% (mRNA-S1+S2-LNP) and 17.16% (mRNA-S1+S2+E-LNP). Similarly, CD3^+^CD8^+^ T cells increased from 4.43% (PBS) and 5.39% (inactivated) to 7.32% (mRNA-S1+S2-LNP) and 7.96% (mRNA-S1+S2+E-LNP). No statistically significant difference was observed between the two mRNA vaccine groups for either CD4^+^ or CD8^+^ T-cell populations (*p* > 0.05), indicating comparable cellular immune activation.

These findings indicate that the multi-epitope mRNA vaccine effectively promoted both helper and cytotoxic T-cell activation, reinforcing its capacity to elicit strong cellular immunity alongside robust humoral responses.

### 3.10. The mRNA Vaccine Demonstrates a Favorable Safety Profile

To evaluate the biosafety of the developed mRNA–LNP formulations, clinical observations, body weight monitoring, and histopathological examinations were performed following immunization.

**Figure 11:**
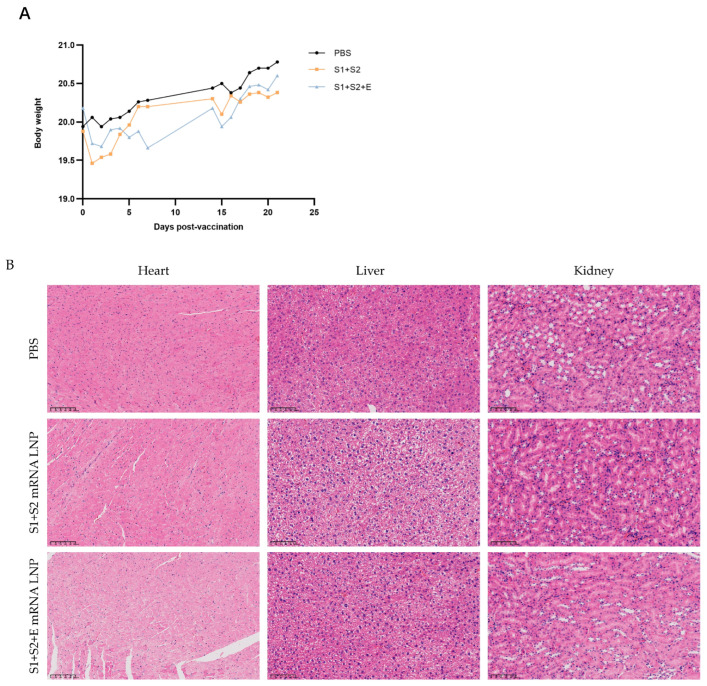
In vivo safety evaluation of PEDV mRNA vaccines. (**A**) Body weight changes of mice following immunization. (**B**) Representative H&E-stained sections of heart, liver, and kidney tissues (scale bar = 100 μm).

Throughout the vaccination schedule, no abnormal behaviors, local swelling, or injection-site redness were observed in any experimental group. As shown in Figure 11A, the body weights of all immunized mice remained stable and comparable to those of the PBS control group (*p* > 0.05), indicating that the vaccine formulations did not induce any observable systemic toxicity or affect normal physiological functions.

Histopathological examination of major organs, including the heart, liver, and kidneys, revealed no evidence of inflammatory infiltration, necrosis, edema, or tissue damage in any group (Figure 11B). The architecture of cardiac muscle fibers, hepatic lobules, and renal tubules remained intact and consistent with normal morphology. No histological differences were observed between vaccinated and control groups.

Collectively, these findings confirm that the PEDV mRNA–LNP vaccines exhibit excellent safety and biocompatibility, causing no detectable adverse effects in mice at the tested doses.

## 4. Discussion

Porcine epidemic diarrhea virus (PEDV) continues to pose a serious threat to the global swine industry, especially in neonatal piglets, highlighting the need for more effective vaccine strategies [47]. Although traditional inactivated and attenuated vaccines have been developed, their protective efficacy remains unsatisfactory due to viral genetic diversity and antigenic drift [48]. Therefore, the development of next-generation vaccines that can induce broad and durable immune protection is of great importance.

In this study, a multi-epitope mRNA vaccine was designed based on immunoinformatics analysis of the PEDV S, M, and N proteins, which contain abundant B- and T-cell epitopes and play essential roles in viral infection and immune recognition. Through the integration of multiple predicted linear B-cell epitopes and dominant T-cell epitopes, a novel antigen (E) was constructed to enhance the immunogenic coverage of PEDV [49]. Compared with conventional single-antigen vaccine designs, this multi-epitope approach may provide broader immunogenicity and improved cross-protective potential, which is particularly important for highly variable viruses.

The stability and efficient translation of the synthesized mRNA constructs observed in this study suggest that the mRNA–LNP delivery system can maintain structural integrity and facilitate effective antigen expression in vivo. This is a critical prerequisite for mRNA vaccine performance, as antigen expression directly determines the magnitude of the immune response. Similar findings have been reported in previous studies, where LNP-based delivery systems enhanced mRNA stability and translation efficiency, supporting their widespread application in vaccine development [50].

The enhanced humoral immune responses observed, particularly in the mRNA-S1+S2+E-LNP group, suggest that the inclusion of the multi-epitope construct (E) contributed to improved antigen recognition and immune coverage. This may be attributed to the increased diversity of epitopes, which facilitates broader B-cell activation and promotes the production of neutralizing antibodies. Similar studies have shown that multi-epitope vaccine designs can significantly enhance antibody responses compared with single-antigen strategies. Therefore, the present findings support the effectiveness of immunoinformatics-guided multi-epitope design in improving vaccine-induced humoral immunity.

The activation of both CD4^+^ helper and CD8^+^ cytotoxic T-cell responses further indicates that the mRNA vaccines are capable of inducing balanced cellular immunity. This is essential for antiviral protection, as CD4^+^ T cells support antibody production while CD8^+^ T cells contribute to the elimination of infected cells. The increased secretion of IL-4 and IFN-γ suggests activation of both Th2 and Th1 pathways, which is consistent with previous reports on mRNA vaccine-induced immune responses [51]. Such a balanced immune profile is considered beneficial for achieving durable and effective protection against viral infections.

The absence of significant adverse effects and histopathological changes indicates that the mRNA–LNP formulations possess a favorable safety profile. This is an important consideration for vaccine development, as safety is a key factor influencing clinical translation. The observed biocompatibility is consistent with previous reports on mRNA–LNP platforms, further supporting their potential as safe and effective vaccine delivery systems [52,53,54].

Compared with previously reported PEDV vaccine strategies, the present study provides several advantages. First, the integration of epitopes derived from multiple structural proteins (S, M, and N) improves antigenic coverage compared with conventional single-antigen designs. Second, the immunoinformatics-guided strategy enables more rational epitope selection, which may enhance immune response efficiency. Third, the use of an mRNA–LNP platform allows flexible antigen design and efficient in vivo delivery, supporting its potential for rapid adaptation to emerging PEDV variants.

It is important to note that this study was conducted in a mouse model, which is widely used for preliminary evaluation of vaccine immunogenicity due to its well-characterized immune system and practical advantages. As an enteric virus, PEDV relies heavily on mucosal immunity—particularly secretory IgA responses—for protection. Therefore, the reliance on systemic immune indicators in this study (e.g., serum IgG and cytokines) may not fully reflect mucosal immune protection, potentially limiting the interpretation of vaccine efficacy and its translational relevance.

In addition, although the multi-epitope design showed promising immunogenicity, the contribution of individual epitopes was not evaluated, limiting a detailed understanding of epitope-specific immune mechanisms. The durability of the immune response was also not assessed, leaving long-term protective efficacy unclear. Furthermore, while the mRNA–LNP system demonstrated favorable stability and delivery efficiency, additional optimization will be required to support large-scale production and practical application. No viral challenge study was performed in this work; therefore, the protective efficacy of the vaccine construct could not be directly evaluated. Therefore, further validation in pigs is essential to assess protective efficacy, mucosal immune responses, and translational potential. Moreover, protein-level validation of antigen expression (e.g., Western blot or antigen-specific detection) was not performed in the present study, which may limit direct confirmation of individual antigen translation despite supportive functional expression evidence.

Taken together, this study provides a preliminary evaluation of a PEDV multi-epitope mRNA vaccine, demonstrating preliminary immune responses and a favorable safety profile, suggesting potential as a next-generation vaccine strategy. These findings support its potential as a next-generation vaccine strategy. Future studies will focus on validating protective efficacy in pigs, optimizing antigen design to address emerging variants, and improving production and delivery systems to facilitate practical application [19].

## 5. Conclusions

In conclusion, this study developed and characterized a novel multi-epitope mRNA vaccine targeting the spike (S) and nucleocapsid (N) proteins of PEDV. Guided by immunoinformatics predictions, multiple dominant B- and T-cell epitopes were rationally integrated to broaden antigenic coverage. The optimized mRNA–LNP formulations exhibited favorable physicochemical properties, high encapsulation efficiency, and effective in vivo expression. Immunization experiments in mice demonstrated that the vaccine construct induced robust PEDV-specific IgG and neutralizing antibody responses, along with strong activation of CD4^+^ and CD8^+^ T-cell responses [55]. Notably, inclusion of the E epitope further enhanced humoral immunity. No apparent adverse reactions or histopathological abnormalities were observed, indicating a favorable safety profile.

In conclusion, this study developed and systematically evaluated a novel multi-epitope mRNA vaccine targeting PEDV by integrating epitopes derived from multiple structural proteins based on immunoinformatics-guided design [56,57]. The mRNA–LNP formulations exhibited favorable physicochemical properties, high stability, and efficient in vivo expression. Immunization in mice demonstrated that the vaccine construct were capable of inducing robust humoral and cellular immune responses, with enhanced antibody responses observed upon inclusion of the multi-epitope construct [55]. In addition, the formulations showed a favorable safety profile in vivo [53,54,55].

These findings provide preliminary evidence supporting the feasibility and potential advantages of multi-epitope mRNA vaccines as a promising strategy for PEDV prevention [58]. Further studies focusing on protective efficacy, immune durability, and validation in target animal models will be necessary to facilitate their translation into practical applications.

## Figures and Tables

**Table 1:** Predicted List of Proteins with Antigenicity and Their Corresponding Antigenicity Probability.

Number	UniProtKB ID	Protein Name	Antigenic Probability	
			VaxiJen	ANTIGENpro
1	P0C6V6	Replicase polyprotein 1a	0.46	0.27
2	P0C6Y4	Replicase polyprotein 1ab	0.46	0.27
3	Q84706	Envelope small membrane protein	0.56	0.11
4	Q91AV1	Spike glycoprotein	0.43	0.53
5	Q07499	Nucleoprotein	0.61	0.92
6	P59771	Membrane protein	0.62	0.22
7	Q91AV0	Non-structural protein 3	0.61	0.13

**Table 2:** Predicted B-cell epitopes of candidate antigens.

Protein	Position	B Cell Epitope	Length	Score
Q91AV1	57–72	KNQGVNSTWYCAGQHP	16	0.6176
	302–316	TIDGVCNGAAVQRAP	15	0.4226
	499–638	ISFVTLPSFNDHSFVNITV SASFGGHSGANLIASDTTINGLSSFCVDTRQFTISLFYNVTNSYGYVSKSQDSNCPFTLQSVNDYLSFSKFCVSTSLLASACTIDLFGYPEFGSGVKFTSLYFQFTKGELITGTPKPLRGV	140	0.5215
	879–896	GVSVYDPASGRVVQKRSF	8	0.5309
	1312–1327	VDLEWLNRVETYIKWP	16	1.2522
P59771	152–168	EGYKVATGVQVSQLPNF	17	0.5581
	174–194	ATTTIVYGRVGRSVNASSGTG	21	0.8136

**Table 3:** Predicted SLA-1–Restricted CTL Epitopes of PEDV Proteins.

Protein	Position	Petides	SLA-1:0101	SLA-1:0401	SLA-1:0801
Q91AV1	176–184	FSDKIYHFY	SB	SB	SB
	206–214	YVYEPTYYM	SB	SB	SB
	352–360	SSDPHLATF	SB	SB	SB
	418–426	LLDAVTINF	SB	SB	SB
	1066–1074	SIDDIYSRL	SB	SB	SB
	1145–1153	QAAPQGLLF	SB	SB	SB
	1251–1259	TLDEILASL	SB	SB	SB
P59771	63–71	SLFDAWASF	WB	SB	SB
	189–197	ASSGTGWAF	WB	SB	SB
	208–216	AVSNPSAVL	SB	WB	WB
Q07499	324–332	SLAPNVAAL	SB	WB	SB
	343–351	LADSYEITY	SB	SB	SB
	359–367	KSDPNVELL	SB	SB	WB

SB denotes strong binders, whereas WB represents weak binders.

## Data Availability

Data supporting the main conclusions of this study are included in the manuscript.

## Supplementary Materials

The following supporting information can be downloaded at: https://www.mdpi.com/article/10.3390/vaccines14050388/s1, The uncropped blots.
