# Supplementary material for: Preliminary Immunogenicity Evaluation of an Immunoinformatics-Guided Multi-Epitope mRNA Vaccine Against Porcine Epidemic Diarrhea Virus
Source: Vaccines (Basel). 2026 Apr 27;14(5):388. doi: 10.3390/vaccines14050388 (PMC13211352; doi:10.3390/vaccines14050388)
Supplement: Supplementary file 1 [file vaccines-14-00388-s001.zip › vaccines-4251623-supplementary.pdf]

Uncropped gel image from Figure 5A in the main text

M 1 2 3 4 5 6

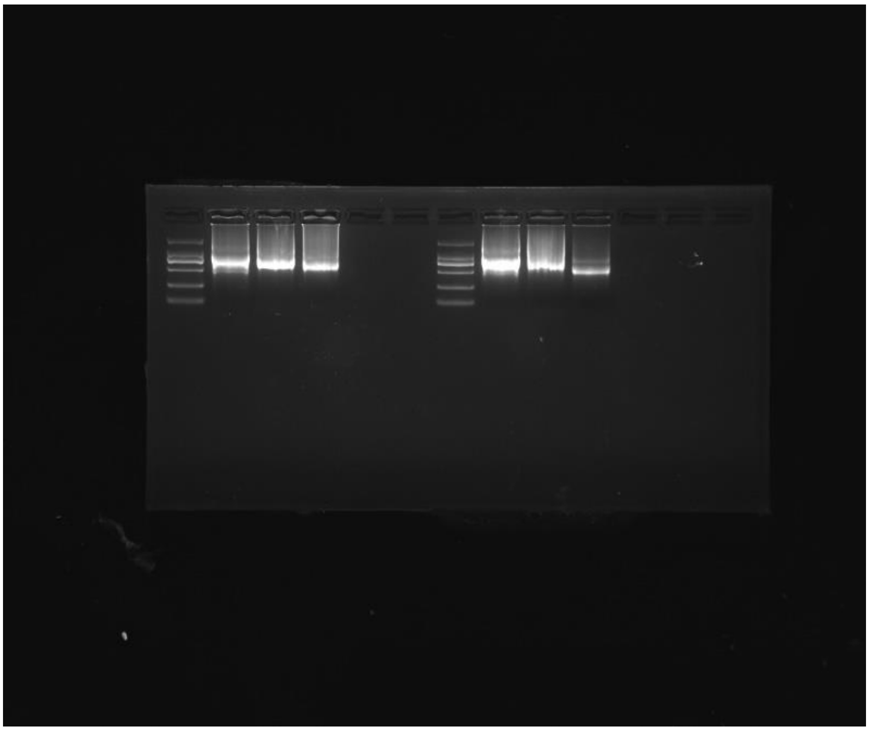

Agarose gel electrophoresis analysis of in vitro–transcribed mRNA encoding S1, S2, and E antigens. What we provide in the main text are lanes 4 to 6, which belong to the products after in vitro transcription. Lanes 1-3 are the products after sedimentation.
